# Supplementary material for: Multicenter validation of a machine learning phase space electro-mechanical pulse wave analysis to predict elevated left ventricular end diastolic pressure at the point-of-care
Source: PLoS One. 2022 Nov 15;17(11):e0277300. doi: 10.1371/journal.pone.0277300 (PMC9665374; doi:10.1371/journal.pone.0277300)
Supplement: S1 File — (DOCX) [file pone.0277300.s001.docx]

**S1– Multivariate Clinical Predictors of Elevated LVEDP**

| Predictor | Odds Ratio | Adjusted p-value |
| --- | --- | --- |
| BMI ≥ 30 | 6.57 (95% CI: 2.49-17.33) | <0.05 |
| Diuretic | 6.12 (95% CI: 2.41-15.52) | <0.05 |
| Female | 4.05 (95% CI: 1.63-10.07) | <0.05 |
| Age ≥ 60 | 1.70 (95% CI: 0.67-4.35) | 0.27 |
| Ejection Fraction < 50% | 1.69 (95% CI: 0.27-10.72) | 0.58 |
| Obstructive CAD | 1.44 (95% CI: 0.54-3.80) | 0.47 |
| Diabetes | 0.89 (95% CI: 0.34-2.30) | 0.81 |
| Hypertension | 0.41 (95% CI: 0.15-1.12) | 0.08 |

*Analysis performed with multivariate logistic regression (fitglm in Matlab) to detect predictors of elevated LVEDP≥25mmHg vs. LVEDP≤12mmHg. N=139 subjects were excluded due to unknown diabetes (N=1) or ejection fraction not measured during catherization (N=138).*
