# Supplementary material for: Multicenter validation of a machine learning phase space electro-mechanical pulse wave analysis to predict elevated left ventricular end diastolic pressure at the point-of-care
Source: PLoS One. 2022 Nov 15;17(11):e0277300. doi: 10.1371/journal.pone.0277300 (PMC9665374; doi:10.1371/journal.pone.0277300)

**S2 – Feature Permutation Importance**

Feature permutation importance was computed by permutating the values of the features across the dataset and observing the resultant effect on performance. The importance was summed within each feature family, which is shown in the figure below as a cumulative sum across the families, reaching the total contribution across the 30 features (set to a normalized value of 1). The most contributive signal-based feature family was PPG indicators, followed by OVG spectral and phase space analysis.


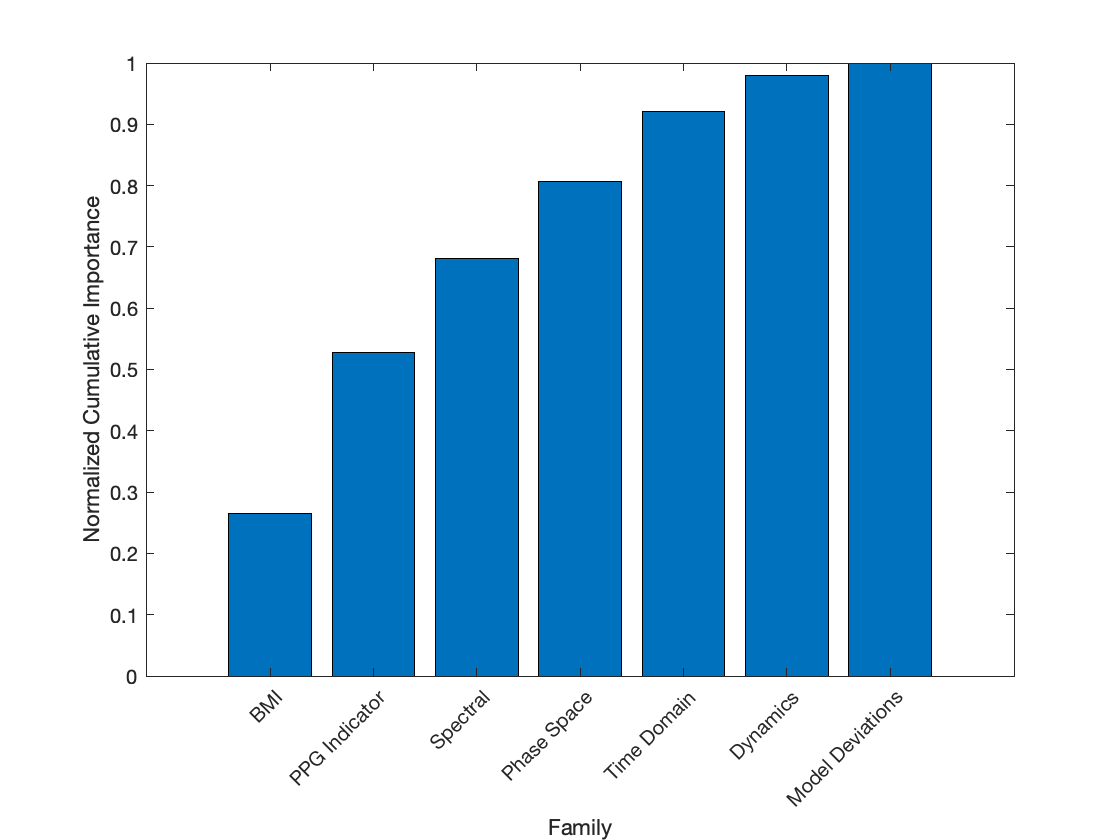

Supplement: S2 File — (DOCX) [file pone.0277300.s002.docx]
