# Supplementary material for: Multicenter validation of a machine learning phase space electro-mechanical pulse wave analysis to predict elevated left ventricular end diastolic pressure at the point-of-care
Source: PLoS One. 2022 Nov 15;17(11):e0277300. doi: 10.1371/journal.pone.0277300 (PMC9665374; doi:10.1371/journal.pone.0277300)
Supplement: S3 File — (DOCX) [file pone.0277300.s003.docx]

**S3 – Features**

Each of the feature families used in the present work is described in the table below, including the characteristics of the signal that is being quantified, as well as the calculation and compression methodology. Specifically, the signal is acquired for a duration of 3.5 minutes, encompassing many cardiac cycles. Features are extracted this duration in three ways:

1. Cycle-by-cycle: The feature is calculated on each cardiac cycle, yielding a distribution of features across the cycles. The feature is then compressed through the calculation of parameters of that distribution, such as mean, median, interquartile range, and percentiles.
2. Longer-duration segment(s): The feature is calculated across longer-duration segment(s) that encompass multiple cardiac cycles, after which the same compression strategy as used for cycle-by-cycle features is applied. However, parameters are limited to central tendency measures (i.e., mean and median).
3. Whole-signal calculation: The feature is calculated using the entire duration of the signal, after which no compression is required.

Below the feature family table are in-depth descriptions of several features.

| **Feature Domain** | **Description** |
| --- | --- |
| Dynamics of the OVG and PPG signals | Features from the dynamical systems domain.  Features calculated using the PPG and OVG signals to characterize the synchronicity using Poincare maps^[[1]](#footnote-1)^.  Feature calculation performed using the whole-signal approach. |
| Spectral properties of OVG and PPG signals | Features quantifying the OVG and PPG signals, including power spectral analysis and time-frequency analysis.  Feature calculation performed using the longer-duration segment approach. |
| Deviations of the OVG signal from subject-specific models | Features that assess the deviation of the OVG signal from subject-specific models.  Feature calculation performed using the cycle-by-cycle approach. |
| Conventional time-domain features and variations of those features | OVG features to quantify both conventional cardiac measurements and variations of those measurements.  Feature calculation performed using the cycle-by-cycle approach. |
| Phase space features | Features calculated using the OVG and PPG in phase space, with the latter requiring phase space reconstructions, comprised of geometric measurements and relationships between physiological loops.  Feature calculation is performed using the longer-duration segment approach for OVG, and the cycle-by-cycle approach for PPG |
| Photoplethysmographic indicators | Features that quantify properties of the PPG in the time domain.  Feature calculation is performed using the cycle-by-cycle approach |

Atrial Depolarization in Phase Space – Phase Space Domain

Atrial depolarization in phase space is populated by coordinates formed by the amplitude of the first and second channel in millivolts at each time point. The maximal atrial depolarization vector is defined by the maximal atrial depolarization amplitude and the isoelectric portion of the signal, which is compared to a reference vector to calculate the angle.

PPG Pulse Base – PPG Indicator Domain

A feature that is extracted from the PPG signal is based on the amplitude of the pulse base, which is the lowest absorption occurring at the minimal blood volume at the end of diastolic phase and beginning of the next systolic cycle. The maximum value of this landmark is captured as a feature, compressed using the 75th percentile from the distribution of the amplitude across the PPG signal. The percentile compression ensures that the feature is robust to any outliers that may occur as a result of artifacts and ambient noise.

Ventricular Repolarization in Bandpass-Filtered Phase Space – Phase Space Domain

A feature which combines multiple domains of signal characterization is an analysis of the ventricular repolarization in a band-pass filtered phase space. Similar analyses on other waveforms have been previously reported^[[2]](#footnote-2)^. Here, the ventricular repolarization waveforms are identified in the time domain, then the signal is band-pass filtered to only preserve frequency content between 70 and 250Hz. Each ventricular repolarization waveform is separated into five segments of equal duration, and a plane is fit to each segment in phase space. The angle between the planes of corresponding segments in consecutive beats is then calculated, which is averaged over all beats in the signal.

1. Fathieh F, Paak M, Khosousi A, et al. Predicting cardiac disease from interactions of simultaneously-acquired hemodynamic and cardiac signals. Comput Methods Programs Biomed. 2021;202. [↑](#footnote-ref-1)
2. Tereshchenko, Larisa G., et al. "Analysis of speed, curvature, planarity and frequency characteristics of heart vector movement to evaluate the electrophysiological substrate associated with ventricular tachycardia." Computers in biology and medicine 65 (2015): 150-160. [↑](#footnote-ref-2)
