# Supplementary material for: Multicenter validation of a machine learning phase space electro-mechanical pulse wave analysis to predict elevated left ventricular end diastolic pressure at the point-of-care
Source: PLoS One. 2022 Nov 15;17(11):e0277300. doi: 10.1371/journal.pone.0277300 (PMC9665374; doi:10.1371/journal.pone.0277300)
Supplement: S4 File — (DOCX) [file pone.0277300.s004.docx]

**S4 – 2x2 Contingency Tables & Additional Statistics**

|  |  | LVEDP from cardiac catheterization | |
| --- | --- | --- | --- |
|  |  | LVEDP ≥ 25 | LVEDP ≤ 12 |
| ML Predictor | LVEDP ≥ 25 | 65 | 83 |
|  | LVEDP ≤ 12 | 14 | 175 |

|  |  |  |
| --- | --- | --- |
|  |  | Healthy Cohort without CV Disease |
| ML Predictor | LVEDP ≥ 25 | 10 |
|  | LVEDP ≤ 12 | 178 |

Negative predictive value (NPV) can also be calculated on the healthy control cohort using the above 2x2 tables, since the threshold on the continuous machine-learned score is held constant throughout. NPV varies depending on disease prevalence, so various values of disease prevalence are shown in the below table. Sensitivity in constant in this table at 82%, as is specificity on the healthy control cohort at 95%, since these statistics do not vary with disease prevalence.

| **Disease Prevalence** | **NPV** |
| --- | --- |
| 10% | 98% |
| 20% | 95% |
| 30% | 92% |
| 40% | 89% |
| 50% | 84% |
| 60% | 78% |
| 70% | 69% |
| 80% | 57% |
| 90% | 37% |

The ROC curve when considering only subjects with elevated LVEDP and healthy control cohort is shown below.

**
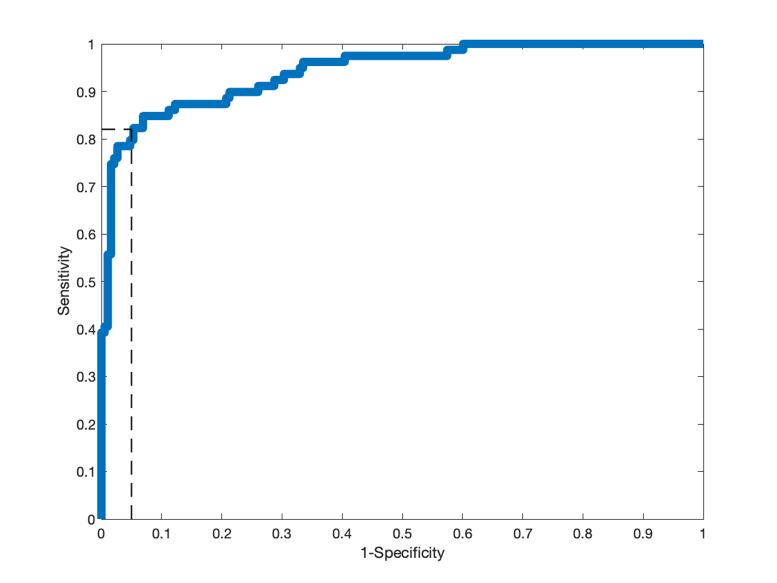
**

Area under the ROC Curve

0.94 (95% CI: 0.90-0.97)

Sensitivity: 0.82 (0.72-0.90)

Specificity: 0.95 (0.90-0.97)
