# Supplementary material for: Multicenter validation of a machine learning phase space electro-mechanical pulse wave analysis to predict elevated left ventricular end diastolic pressure at the point-of-care
Source: PLoS One. 2022 Nov 15;17(11):e0277300. doi: 10.1371/journal.pone.0277300 (PMC9665374; doi:10.1371/journal.pone.0277300)
Supplement: S5 File — (DOCX) [file pone.0277300.s005.docx]

**S5 – Propensity Matching**

Matching was performed using the MatchIt R package^[[1]](#footnote-1)^, where the propensity scoring was done using a generalized linear model (GLM, as reported in the main manuscript) and a random forest, and further Mahanobis distance was used as a direct measurement of similarity (score-less). In each case, nearest neighbor matching was performed to create the most similar subgroups of elevated and non-elevated LVEDP based on gender and age.

The GLM performance was reported in the manuscript and is reproduced here for context. All the matching methods yield similar performances, with GLM in the middle, Random Forest slightly lower, and Mahanobis slightly higher.

| Propensity Matching Method | AUC in Matched Data |
| --- | --- |
| GLM | 0.79 (95% CI: 0.72, 0.86) |
| Random Forest | 0.77 (95% CI: 0.69, 0.84) |
| Mahanobis | 0.82 (95% CI: 0.76, 0.89) |

1. Ho, D. E., Imai, K., King, G., & Stuart, E. A. (2011). MatchIt: Nonparametric Preprocessing for Parametric Causal Inference. Journal of Statistical Software, 42(8). doi:10.18637/jss.v042.i08 [↑](#footnote-ref-1)
