# Supplementary material for: Multicenter validation of a machine learning phase space electro-mechanical pulse wave analysis to predict elevated left ventricular end diastolic pressure at the point-of-care
Source: PLoS One. 2022 Nov 15;17(11):e0277300. doi: 10.1371/journal.pone.0277300 (PMC9665374; doi:10.1371/journal.pone.0277300)
Supplement: S7 File — (DOCX) [file pone.0277300.s007.docx]

**S7 – Enrolling Healthcare Centers**

The primary requirement in site selection was that the site have a well-established research program. A secondary requirement as that the sites have high catheterization lab volumes to support study enrollment. There were no special subject characteristics for each center; subjects must simply meet the study inclusion and exclusion criteria. Furthermore, our sites encompass both clinic environments as well as hospitals, specifically designed to increase diversity of enrollment, and for broad geographic representation.

| Center | Number of Study Subjects Enrolled |
| --- | --- |
| Atlanta Heart Specialists  *Cumming, GA* | 13 |
| LeBauer-Brodie Center for Cardiovascular Research  *Greensboro, NC* | 116 |
| New Hanover Regional Medical Center  *Wilmington, NC* | 119 |
| The Rochester General Hospital  *Rochester, NY* | 28 |
| Bryan Heart  *Lincoln, NE* | 124 |
| Piedmont Healthcare  *Atlanta, GA* | 48 |
| Austin Heart (Round Rock Office)  *Austin, TX* | 58 |
| AdventHealth Tampa  *Tampa, FL* | 5 |
| Cardiovascular Institute o/t South (Houma)  *Houma, LA* | 12 |
| Cardiovascular Institute o/t South (Lafayette)  Lafayette, LA | 19 |
| Jackson Heart Clinic  *Jackson, MS* | 66 |
| Oklahoma Heart Hospital  *Oklahoma City, OK* | 8 |
| WellStar Research Institute  *Marietta, GA* | 14 |
| Cardiology Associates Research, LLC  *Tupelo, MS* | 53 |
| Medical University of South Carolina  *Charleston, SC* | 1 |
