# Supplementary material for: Multicenter validation of a machine learning phase space electro-mechanical pulse wave analysis to predict elevated left ventricular end diastolic pressure at the point-of-care
Source: PLoS One. 2022 Nov 15;17(11):e0277300. doi: 10.1371/journal.pone.0277300 (PMC9665374; doi:10.1371/journal.pone.0277300)
Supplement: S8 File — (DOCX) [file pone.0277300.s008.docx]

**S8 – Inclusion and Exclusion Criteria**

Inclusion criteria:

1. Age > 18 years.
2. Symptoms suggestive of obstructive CAD.
3. Scheduled to undergo cardiac catheterization with coronary angiography.
4. Understand the requirements of the study and to provide written informed consent.
5. Current rhythm of sinus rhythm.

Exclusion criteria:

1. Prior documented history of myocardial infarction (MI).
2. Suspected acute myocardial infarction (AMI) at current presentation.
3. Prior coronary artery bypass grafting (CABG).
4. Prior heart valve repair or replacement.
5. History of atrial or ventricular arrythmia.
6. Diagnosis of infiltrative myocardial disease (amyloid, sarcoid, right ventricular dysplasia or others).
7. Presence of cardiac implantable electronic device (CIED) including implantable cardioverter defibrillator (ICD), pacemaker (PM), cardiac resynchronization therapy devices (CRT), implantable loop recorders or other devices.
8. Implantable neuro-stimulators.
9. Congenital heart disease.
10. Pregnant or breast feeding.
11. Currently prescribed any Type IA, IC or III antiarrhythmics.
12. History of amiodarone therapy.
13. Clinically significant chest deformity (e.g., pectus excavatum or pectus carinatum).
14. Breast implants.
15. Neuromuscular Disease if the condition results in tremor or muscle fasciculations.
