# Supplementary material for: Multicenter validation of a machine learning phase space electro-mechanical pulse wave analysis to predict elevated left ventricular end diastolic pressure at the point-of-care
Source: PLoS One. 2022 Nov 15;17(11):e0277300. doi: 10.1371/journal.pone.0277300 (PMC9665374; doi:10.1371/journal.pone.0277300)
Supplement: S9 File — (DOCX) [file pone.0277300.s009.docx]

**S9 – Placement of the OVG Electrodes and PPG Sensor**

*Note that the individual in the image below is a model (not a study subject) who has provided a photographic release for use of his image.*

| 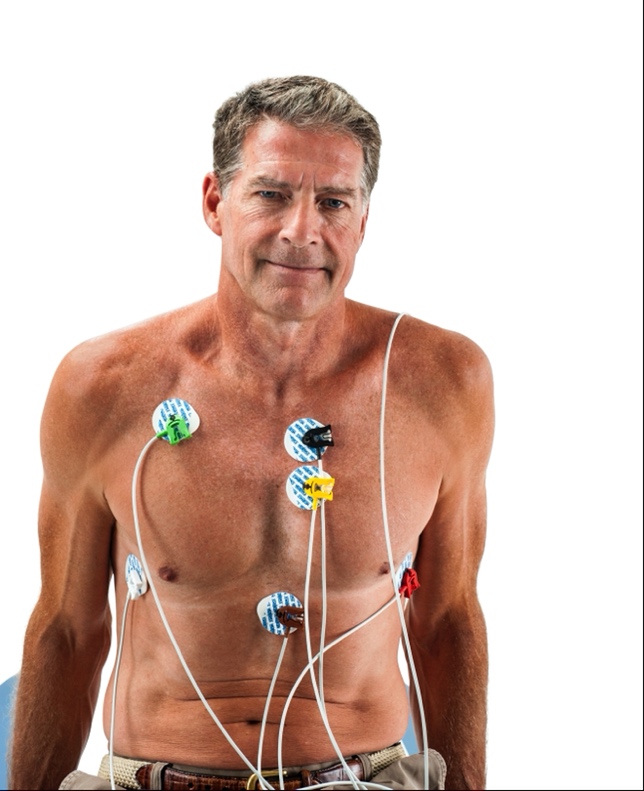 | 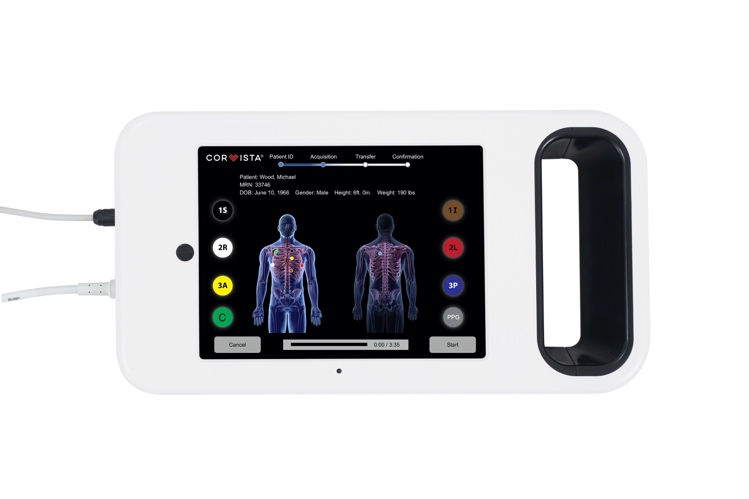 |
| --- | --- |
|  | 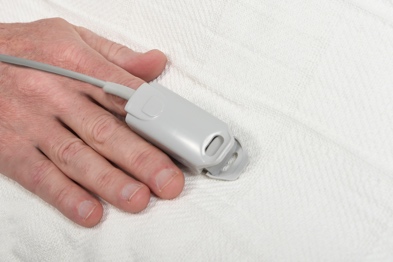 |
| **Electrode** | **Position** |
| White | Right anterior axillary line, in 5^th^ intercostal space |
| Red | Left anterior axillary line, in 5^th^ intercostal space |
| Black | Left sternal border, in 1^st^ intercostal space |
| Brown | Left sternal border, below the sternum and just lateral to the xiphoid process |
| Blue | On the back, directly aligned to the opposite yellow electrode, to the left of the spine. |
| Yellow | Left sternal border, in the 3^rd^ intercostal space |
| Green | Right upper quadrant and right axillary line, in the 2^nd^ intercostal space. |
