# Supplementary material for: Multicenter validation of a machine learning phase space electro-mechanical pulse wave analysis to predict elevated left ventricular end diastolic pressure at the point-of-care
Source: PLoS One. 2022 Nov 15;17(11):e0277300. doi: 10.1371/journal.pone.0277300 (PMC9665374; doi:10.1371/journal.pone.0277300)
Supplement: S10 File — (DOCX) [file pone.0277300.s010.docx]

**S10 – Model Specifications**

Unless otherwise noted in the table, the hyperparameters for the algorithms were set to the default value.

| **Number** | **Algorithm** | **Training Data** | **Number of Features** | **Hyperparameters** |
| --- | --- | --- | --- | --- |
| 1 | Random Forest | LVEDP≤12 and LVEDP≥20 | 180 | Maximum Tree Depth = 3  Minimum Samples Per Leaf = 1  Number of Trees = 500  Bootstrapping = True |
| 2 | Elastic Net | LVEDP≤12 and LVEDP≥20 | 194 | Alpha = 0.003  Fit Intercept = True  L1_ratio = 0  Normalize = False |
| 3 | Random Forest | LVEDP≤12 and LVEDP≥20 | 95 | Maximum Tree Depth = 3  Minimum Samples Per Leaf = 1  Number of Trees = 100  Bootstrapping = True |
| 4 | Extreme Gradient Boosting | LVEDP≤12 and LVEDP≥25 | 162 | Learning Rate = 0.5  Maximum Tree Depth = 3  Minimum Child Weight = 3  Number of Trees = 100  Regularization Alpha = 0.1 |
| 5 | Extreme Gradient Boosting | LVEDP≤12 and LVEDP≥25 | 194 | Learning Rate = 0.3  Maximum Tree Depth = 7  Minimum Child Weight = 3  Number of Trees = 100  Regularization Alpha = 0.5 |
| 6 | Extreme Gradient Boosting | LVEDP≤12 and LVEDP≥25 | 134 | Learning Rate = 0.5  Maximum Tree Depth = 7  Minimum Child Weight = 5  Number of Trees = 100  Regularization Alpha = 0.1 |
| 7 | Elastic Net | LVEDP≤12 and LVEDP≥25 | 194 | Alpha = 0.003  Fit Intercept = True  L1_ratio = 0  Normalize = True |
| 8 | Extreme Gradient Boosting | LVEDP≤12 and LVEDP≥25 | 152 | Learning Rate = 0.3  Maximum Tree Depth = 5  Minimum Child Weight = 3  Number of Trees = 100  Regularization Alpha = 0.3 |
| 9 | Elastic Net | LVEDP≤12 and LVEDP≥25 | 122 | Alpha = 0.01  Fit Intercept = True  L1_ratio = 0.1  Normalize = True |
| 10 | Random Forest | LVEDP≤12 and LVEDP≥25 | 179 | Maximum Tree Depth = 3  Minimum Samples Per Leaf = 1  Number of Trees = 500  Bootstrapping = True |
| 11 | Random Forest | LVEDP≤12 and LVEDP≥25 | 89 | Maximum Tree Depth = 7  Minimum Samples Per Leaf = 1  Number of Trees = 50  Bootstrapping = True |
| 12 | Extreme Gradient Boosting | LVEDP≤12 and LVEDP≥25 | 119 | Learning Rate = 0.3  Maximum Tree Depth = 7  Minimum Child Weight = 3  Number of Trees = 10  Regularization Alpha = 0.3 |
| 13 | Elastic Net | LVEDP≤12, LVEDP≥25, healthy subjects | 122 | Alpha = 0  Fit Intercept = False  L1_ratio = 0.1  Normalize = False |

**
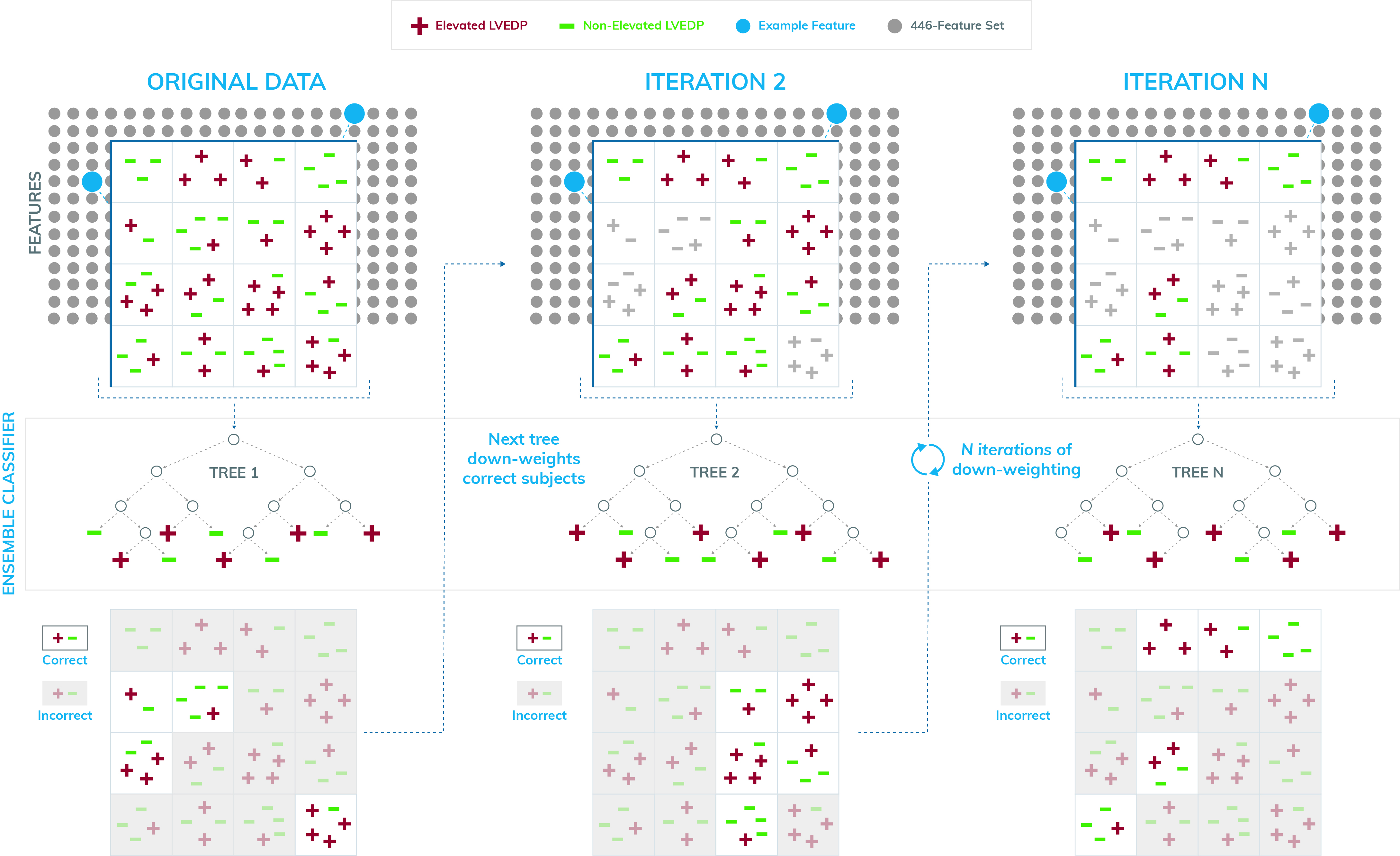
**

The process for training an Extreme Gradient Boosting (XGB) model is shown in the image above, using an example scenario with only two features. The first tree is exposed to all the training data without any weighting, and the tree’s performance is quantified on the training data. The tree will get some subjects correct, and some incorrect, and that information is passed through to the second iteration, where subjects that were already predicted correctly are downweighted in order to prioritize subjects that were previously predicted incorrectly. The iterative process continues over *N* trees, after which all the trees are included in the ensemble.
