# Supplementary material for: Multicenter validation of a machine learning phase space electro-mechanical pulse wave analysis to predict elevated left ventricular end diastolic pressure at the point-of-care
Source: PLoS One. 2022 Nov 15;17(11):e0277300. doi: 10.1371/journal.pone.0277300 (PMC9665374; doi:10.1371/journal.pone.0277300)
Supplement: Attachment — Submitted filename: Response to Reviewers.pdf [file pone.0277300.s012.pdf]

Dear Reviewer #1,

We greatly appreciate the comments towards improving the quality of our work. Please see a response to each of your comments below.

Comment #1: The authors can provide more clear description for the definition of the indices used as features (perhaps provide some figure illustrations).

We thank the reviewer for suggesting a clearer description of the features used. In this context, please find Figure 3, and Supplement Figure 3 (both reproduced below) that are provided to describe and illustrate example features used in the algorithm. Figure 3 illustrates 4 features used in the algorithm, which are described as: 1) the variation in the atrial depolarization duration is a feature extracted from the OVG signal in the time domain, 2) quantification of the atrial depolarization vector in phase space, 3) quantification of PPG pulse base amplitude and 4) ventricular repolarization in band-pass filtered phase space. Supplement Section 3 defines 6 feature families that include all features contributing to the algorithm.

A description of each are provided in the methods and results, and the clinical translation of the features (physiologic representation of atrial and ventricular depolarization and repolarization in heart failure) are provided in the discussion.

To provide addition details, please find an expansion to Supplement Section 3 with the following:

> Each of the feature families used in the present work is described in the table below, including the characteristics of the signal that is being quantified, as well as the calculation and compression methodology. Specifically, the signal is acquired for a duration of 3.5 minutes over consecutive cardiac cycles. Features are extracted this duration in three ways:
>
> 1. Cycle-by-cycle: The feature is calculated on each cardiac cycle, yielding a distribution of features across the cycles. The feature is then compressed through the calculation of parameters of that distribution, such as mean, median, interquartile range, and percentiles.
>
> 2. Longer-duration segment(s): The feature is calculated across longer-duration segment(s) that encompass multiple cardiac cycles, after which the same compression strategy as used for cycle-by-cycle features is applied. However, parameters are limited to central tendency measures (i.e., mean and median).
>
> 3. Whole-signal calculation: The feature is calculated using the entire duration of the signal, after which no compression is required.

Comment #2: Since this is a multi-center study, the authors can provide descriptions about the chosen centers. For example, why choosing these centers? Any special subject characteristic for each center?

The primary requirement in site selection was that the site have a well-established clinical and research program. A secondary requirement as that the sites have high catheterization lab volumes to support study enrollment. There were no special subject characteristics for each center; subjects must simply meet the study inclusion and exclusion criteria. Furthermore, our sites encompass both outpatient clinical environments as well as hospitals and were specifically chosen to increase the diversity of enrollment and for broad geographic representation.

Please find the paragraph above added to Supplement Section 7 to provide additional details in the selection of enrollment centers.

Dear Reviewer #2,

We greatly appreciate the comments towards improving the quality of our work. Please see a response to each of your comments below.

Introduction

Comment #1: It would be nice to include some literature reviews on any previous studies predicting LVEDP using non-invasive measurement, if any.

Thank you for suggesting additional context and previous studies using LVEDP. Similar to your comment and suggested changes between the introduction and discussion sections, please find lines 101-115 of the Introduction amended (below) to include previous studies predicting LVEDP using non-invasive measurements such as echocardiography.

> In one possible application, while systolic dysfunction is characterized by reduced ejection fraction, additional modalities are required to adjudicate dysfunction that is limited to diastole, with the aim of estimating left ventricular (LV) filling pressures. Left ventricular end diastolic pressure (LVEDP) is of distinct interest. The measurement of LVEDP, whether in the presence of reduced or preserved ejection fraction is complex and commonly characterized by multimodality diagnostic imaging. For example, elevation in Brain Naturetic Peptide (BNP) (2,3) and fixed ratios based on echocardiography (spectral Doppler and Tissue Doppler derived E/e') (4) are used to classify if left atrial pressure is elevated or not. Several recent studies have aimed to predict diastolic dysfunction (i.e., intracardiac pressure elevation) using ML approaches, such as from CNN analysis of echocardiographic beat variability (5) and clustering of echocardiographic markers to understand the patterns of diastolic dysfunction across patients with symptomatic CVD (6). While such developments are promising in the characterization of myocardial function, the prediction of LV pressure elevation as a binary classification (elevated or not elevated) across a spectrum of LV pressures that can be used to guide downstream testing and treatment is of value.

Results

Comment #2: Are all the results shown in the results section using the ensembled model?

Yes, all the results presented in the results section are using the ensembled model. Our validation plan was a single assessment of the algorithm performance on the blinded validation cohort. The sole assessment was chosen to nullify any multiplicity issues. Therefore, the validation dataset was assessed only once, using the ensembled model.

Please find lines 191-192 added to the Results section to ensure that the readership understands how the results were generated:

All results (primary and secondary) used the ensembled model as a single assessment of algorithm performance on the blinded validation cohort.

Discussion

Comment #3: The discussion should focus on the results presented in this study, and the literature reviews should be moved to the introduction.

We appreciate this feedback, and we have moved this discussion of the literature to the introduction. Please refer to the response to comment #1 above.

Comment #4: In the limitation, the authors mentioned that study subjects taking diuretics might affect the specificity. Have the authors tried to remove those subjects and rerun the model to test that hypothesis?

We thank the reviewer for the valuable feedback.

As noted in Table 1, 36 of the 258 subjects with non-elevated LVEDP were taking a diuretic. The specificity in this subgroup was 64%, and in the subgroup not taking a diuretic, 68% (p=0.57 for comparison). The specificity in the overall population was 68%. The reduction in specificity in the diuretic subgroup was not significant when considering the small number of subjects in this subgroup to affect the overall specificity. Therefore, while the point-estimate of specificity in the diuretic subgroup was lower than that in the non-diuretic subgroup, the diuretic subgroup is not sufficiently sized to draw any statistical conclusions on differences in specificity.

Please find the limitations section, lines 338-344, updated to reflect the additional analysis above.

> 'We identified 14% (36/258) of subjects with non-elevated LVEDP were taking a diuretic at the time of enrollment that may have impacted the performance of the ML predictor. When compared to overall study population the specificity was similar within this cohort (68% vs 64%, p=0.57) and when the analysis was re-run when excluding this cohort, there was no difference in overall specificity. While we contend that diuretics are an important factor when considering the measurement and prediction of LVEDP, the small number of subjects in this group does not permit us to determine its impact on performance within the study population as presented.'

Comment #5: The authors should add the study populations are patients likely to have CAD to the limitation.

We agree that it's important to capture this aspect of the population in the limitations section, and have added it in the revised manuscript in lines 352-361 as the following:

Our study population is intrinsically limited by the recruitment methodology, which was subjects referred to left heart catheterization for assessment of obstructive CAD using coronary angiography, and specifically the subgroup where the treating physician chose to measure the LVEDP. We employed this study methodology to ensure that subjects had a catheterization-confirmed elevated LVEDP, but at the limitation of subjects referred for the evaluation of obstructive CAD. While this may introduce sample bias, we found significant CAD in only 38% of the overall study cohort, a higher incidence of obstructive CAD was observed in subjects with non-elevated LVEDP compared to those with elevated LVEDP (43% vs 24%). Upon subgroup analysis, there was difference in algorithmic performance among those with or without obstructive CAD.

Comment #6: The authors should also add the possibility of overfitting to the limitation unless they can justify the large number of features they used in the study.

We agree that it's useful to address the possibility of overfitting.

The use of an ensemble does not increase the likelihood of overfitting, but rather mitigates it by eliminating the bias associated with the selection of a single model. Therefore, the possibility of overfitting is analyzed from the perspective of each constituent model.

First, as shown in S10, each model is exposed to an average of 149 features (with a range of 89-194). Secondly, as the model hyperparameters in S10 demonstrate, the models were conservatively designed to mitigate the possibility of overfitting.

Specifically, 4 of the models were Random Forest, which intrinsically limit overfitting by only allowing each component tree access to the square root of the total number of features, and by bootstrap sampling training subjects so that every component tree only has access to a subset of the entire training set. Overfitting was additionally controlled through the use of the maximum tree depth hyperparameter. Deep trees with many splits increases the likelihood of overfitting, and therefore the depth was limited to 3 in most of the models, with the last (which had access to the least number of features) permitted to proceed to a depth of 7.

Second, 4 of the models were Elastic Net, which is a linear model capable of regularization. The linear nature of the model restricts its ability to capture complex interactions between the features. 3 of the 4 Elastic Net models were also regularized (with alpha = 0.003 or 0.01), which limits the ability of the model to rely on any particular small subset of features.

Third, the remaining 5 models were Extreme Gradient Boosting (XGB), which is a boosted tree model. Learning rate, which is step size shrinkage to make boosting more conservative, was set from 0.3-0.5 across the models. Maximum tree depth was also set conservatively to 3-7. Minimum child weight also controls tree partitioning, and was set

to non-zero values (3-5). Regularization alpha was enabled (0.1-0.5) to add L1 regularization to the weights.

Finally, the ultimate test of overfitting is the performance on unseen blinded data, which yielded a high-performing AUC of 0.81.Through the analysis of the algorithm, and the performance on unseen blinded data, overfitting did not occur.

We have modified the limitation section to address overfitting. Please find the following revision on lines 362-453:

Overfitting, and conversely generalizability, are critical aspects of machine learning and when a large number of features are used for model development. The use of an ensemble, as is the case, does not increase the likelihood of overfitting but rather mitigates it by eliminating the bias associated with the selection of a single model. Therefore, the possibility of overfitting should be analyzed from the perspective of the constituent models. Firstly, as shown in S10, each model is exposed to an average of 149 features (with a range of 89-194). Secondly, as the model hyperparameters in S10 demonstrate, the models were conservatively designed to mitigate the possibility of overfitting. For example, four of the models were Random Forest, which intrinsically limit overfitting by only allowing each component tree access to the square root of the total number of features, and by bootstrap sampling training subjects so that every component tree only has access to a subset of the entire training set. Overfitting was additionally controlled through the use of the maximum tree depth hyperparameter. Deep trees with many splits increases the likelihood of overfitting, and therefore the depth was limited to 3-7. Other model types (Elastic Net and XGBoost) were also designed conservatively. Finally, the ultimate test of overfitting is the performance on unseen blinded data, which yielded a high-performing AUC of 0.81. In conclusion, through the analysis of the algorithm, and the performance on unseen blinded data, overfitting did not occur.

Methods

Comment #7: The study population are patients that are likely to have CAD, which seems to be a biased dataset to predict LVEDP. If the aim is being able to predict the LVEDP values for just patients who are likely to have CAD, then this would be acceptable, but if the aim is to predict LVEDP for a wider range of populations, then it would make sense to include other patients irrelevant to having CAD.

The selection of the population is a critical decision and we agree that this is a potential limitation of our study and an important factor when interpreting the generalizability of our results.

On post-hoc analysis, a majority 62% of study subjects did not have obstructive CAD, among which 23% had elevated LVEDP$\geq$25 mmHg. This incidence of non-obstructive CAD at the time of cardiac catheterization among patients referred for the evaluation of

CAD is important and consistent with the population-based data by Patel et al. (NEJM 2010) . As such, our study cohort is mixed, those with and those without obstructive CAD. Upon sub-group analysis (shown in Figure 7, reproduced below) supports that the ML predictor was consistent across sub-populations. While this is exploratory it suggests that there are signals for the generalizability of our results to symptomatic patients without CAD.

Subgroup	AUC (95% C.I.)	N	p-value
Age < 60	0.85 (0.76, 0.93)	119	0.2786
Age >= 60	0.79 (0.71, 0.86)	218	
BMI < 30	0.71 (0.56, 0.86)	161	0.6412
BMI >= 30	0.75 (0.67, 0.83)	176	
Diabetic	0.80 (0.71, 0.90)	102	0.9191
Non-diabetic	0.81 (0.74, 0.88)	234	
Women	0.80 (0.73, 0.88)	152	0.7285
Men	0.78 (0.69, 0.88)	185	
Non-obstructive CAD on angiography	0.83 (0.76, 0.89)	208	0.3060
Obstructive CAD on angiography	0.76 (0.65, 0.87)	129	
Ejection Fraction <= 50%	0.44 (0.11, 0.77)	13	0.0349
Ejection Fraction > 50%	0.85 (0.78, 0.92)	186	
Caucasian	0.80 (0.74, 0.87)	280	0.9776
Black or African American	0.80 (0.67, 0.94)	44	
Sites enrolling less than 50 subjects	0.80 (0.73, 0.87)	230	0.5496
Sites enrolling more than 50 subjects	0.83 (0.73, 0.93)	107	

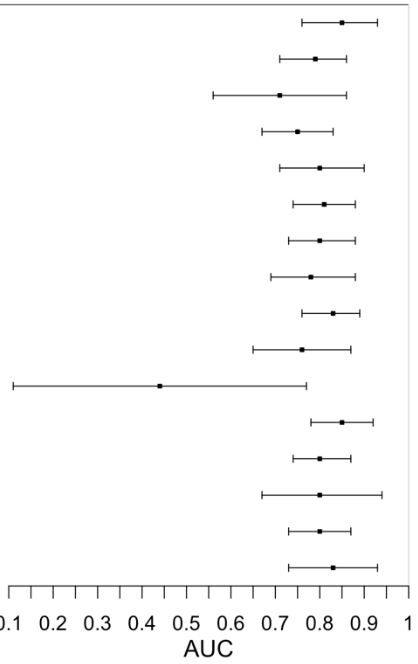

We aim to analyze real world data once the ML device is available for clinical utilization and plan to report the results in a follow up paper.

Comment #8: Line 336: Is it without CVD or CAD? If it is CVD, then which diseases are considered here? As CVD is a very wide category.

CVD is correct – asymptomatic subjects, with risk factors controlled to minimize the chance of undetected CVD of any kind were used for development purposes only (were not included in the validation cohort).

Comment #9: Is the validation group also patients likely to have CAD?

The development cohort was composed of two subgroups: 1) symptomatic patients referred to cardiac catheterization for evaluation of CAD, and who also have a measured LVEDP, and 2) asymptomatic subjects without CVD (see response to Methods Comment #2). The validation cohort was recruited from the exact same population as development cohort – symptomatic patients referred to cardiac catheterization for the evaluation of

CAD. Therefore, yes, the validation group is also composed of patients at suspicion of obstructive CAD.

Comment #10: What type of catheter was used to measure LVEDP, specs?

The choice of catheter to measure the LVEDP was left to the discretion of each interventional cardiologist.

Comment #11: Line 353: it says the following 4 categories, but there are 5 listed.

Thank you, that was a typo; it has been corrected in the revised manuscript.

Comment #12: Line 368: what are the symptoms are considered here for HF

Thank you for identifying a misstatement of the symptoms. These are the subjects from the validation cohort (referred to cardiac catheterization), and the text have been clarified to remove the mention of heart failure on lines 522-524:

> 'Bayesian analysis to determine the post-test (i.e., posterior) probability of the machine-learned predictor based on varying the pre-test probability (i.e., low, intermediate, and high prior probability of elevated LV filling pressures) among symptomatic patients.'

Comment #13: From Line 414 – 420, please give some specific numbers for the cut-offs, e.g. high-frequency noise above XXX Hz?? Maximum measurable value XXX?

The manuscript text has been modified with additional information on SNR. However, the signal quality assessment has been described in detail in a previous publication by our group (Fathieh 2021 discussed in Supplement Section 5).

Please find the modifications below and corresponding line numbers to address SNR:

> Line 576: A SNR of 57 was considered acceptable for powerline noise, and of 19 for high frequency noise.
> Line 582-583: SNR is not applicable to this score because the occurrence is transient.

Comment #14: Could you give a bit more details on how the features are calculated? I imagine you have both OVG and PPG signals with multiple cardiac cycles. Do you calculate the features cycle by cycle? If so, how do you get the final subject level features based on the cycle features? Do you calculate the features over the whole recorded signal? If so, what is the time window you use for each recording? If the time window is constant, then how many seconds? If not, please justify.

The features are extracted in an identical, automated manner across all signals. As mentioned in the Acquisition System Description, we will clarify to provide additional

explanation. The acquisition duration time was 3.5 minutes; therefore, as you mentioned, we have many cardiac cycles per subject.

> Line 532: Signal data was acquired for 3.5 minutes.

In general, the duration of the recording is managed in one of three ways during feature extraction. First, calculating the features on a cycle-by-cycle basis, and statistically compressing across the cycles using the distribution of calculated values across the cycles. Compression can be performed using a variety of distribution parameters, including mean, median, interquartile range, and percentiles. For instance, the PPG Pulse Base feature described in S3 is calculated on a cycle-by-cycle basis, and is compressed through the calculation of the 75th percentile. Second, features are calculated in a series of longer duration segments, after which the same compression strategy is applied (though with parameters limited to central tendency measures, i.e., mean/median). Third, features are calculated using the entire signal, after which no compression is required. S3 has been augmented with this additional information.

Please note, reviewer #1 (comment #1) posed a similar question and the response provided there is included below and added to Supplement Section 3:

> Each of the feature families used in the present work is described in the table below, including the characteristics of the signal that is being quantified, as well as the calculation and compression methodology. Specifically, the signal is acquired for a duration of 3.5 minutes, encompassing many cardiac cycles. Features are extracted this duration in three ways:
> 1. Cycle-by-cycle: The feature is calculated on each cardiac cycle, yielding a distribution of features across the cycles. The feature is then compressed through the calculation of parameters of that distribution, such as mean, median, interquartile range, and percentiles.
> 2. Longer-duration segment(s): The feature is calculated across longer-duration segment(s) that encompass multiple cardiac cycles, after which the same compression strategy as used for cycle-by-cycle features is applied. However, parameters are limited to central tendency measures (i.e., mean and median).
> 3. Whole-signal calculation: The feature is calculated using the entire duration of the signal, after which no compression is required.

Comment #15: Could you provide a list of features used in the appendix? And how they are calculated?

Supplement section 3 contains the families of features used, and now has been augmented with additional information to better describe the calculation (per Comment #14 above).

Comment #16: Is the outlier detection for the cycle level features or in-between subjects as well?

The outlier detection is performed on feature values for each subject to determine if the subject is inlying or outlying. Figure 1 visualizes the flow of subjects from the complete initial population to the study population and includes the identification of outlying subjects (N=49) that were therefore excluded from the analysis.

The text has been modified on line 606 to ensure that it is clear to the reader:

> 'Mathematically outlying subjects were identified based on the signal's feature values using the Isolation Forest algorithm (31).'

Comment #17: The rationale for choosing the 13 machine-learning models is unclear and how the hyper-parameters were chosen is also unclear. The authors claim that the ensemble outputs are intended to outperform any single model, but would they be able to provide some results showing that?

As described in the methods section (Step 4: Machine Learned Model Optimization), the 13 models were selected to capture a diversity of models, encompassing both the ML algorithm itself (Random Forest, Extreme Gradient Boosting, and Elastic Net), training data (with disease-negative always defined as LVEDP≤12mmHg, as compared to either LVEDP≥20mmHg or 25mmHg, and including or excluding healthy subjects), and the features available to the model.

All models exhibited individual predictivity through cross-validation within the development dataset only (the validation cohort was reserved in a blinded manner for only a single test using the final ensemble). Through the diversity provided by varying all these properties, we intended to minimize the likelihood of overfitting the development dataset through the reliance on spuriously predictive patterns that may be present in only one combination, but not another. Further, ensembling removes a decision to select a single model out of the 13 candidates; doing so is a manual step likely to be contaminated by human bias.

Given that our validation plan was strictly a single evaluation on the validation dataset, we cannot state that the ensemble of 13 models is the ideal solution, and that it would have outperformed any single constituent model. However, based on the logic that we outlined in the preceding paragraph, we believe that this is the best strategy that we could have taken, based on the stringency of our validation plan. Specifically, the ensemble approach is intended to on-average outperform any single model that we may have selected from the pool of 13.

We appreciate this question and have updated the manuscript (Step 4: Machine Learned Model Optimization) with clarifications on our methodology that we've described in our response.

Please see the revised lines 615-622 here:

‘Each of the 13 models were individually performant based on cross-validation within the development data (S11), but represent unique analyses of LVEDP assessment. To capture the diversity of each model in a final single prediction, which is intended to eliminate the bias associated with the selection of a single model and thus reduce the likelihood over overfitting on the development data, the 13 model were amalgamated into a single predictive ensemble. The ensemble, composed of an average of the normalized outputs from the constituent models, is intended to on-average outperform any model that we may have selected from the pool of 13 when applied to new data (35).’

Comment #18: In terms of training the model, what type of training was used? Cross-validation?

The models were trained using cross-validation within the development dataset, which has been clarified in the text as described above in Methods question 11.

Comment #19: Can the author provide some information about what they did to prevent overfitting the models?

Please see our response to Comment #17.

Comment #20: Can the author provide some details on how the 95% CIs were calculated?

The CIs for AUC were calculated with De Long’s method. Clopper Pearson was used for sensitivity and specificity calculations. The CIs for the simulation were based on the performance at the 2.5th and 97.5th percentile performance across the simulation iterations.

Please see the modified text on line 637 for AUC, sensitivity and specificity:

‘CIs were calculated using De Long’s method for AUC, and Clopper Pearson for sensitivity and specificity.’

Please see the modified text on lines 669-671 for the simulation:

‘The simulation was repeated for 1000 iterations with the values of the performance statistics averaged and confidence intervals calculated using the distribution of the statistics over the iterations (i.e., values at 2.5th and 97.5th percentiles).’

General Questions

1. Have you investigated the correlations between features?

   It is possible that features may be correlated, and we haven’t investigated this in detail. However, while assumption of non-collinearity is critical for ordinary linear regression (OLR), that assumption is not made for the algorithms included in our ensemble (Elastic

Net, Random Forest and XGBoost). Like OLR, Elastic Net is also a linear model, however Elastic Net is distinguished by the use of regularization, and thus can compensate for correlated features.

Figures

2. Figure 8: could you add what each colour and lines represent?

   We appreciate your comment on this figure – it's now been updated with additional information. Please see the new Fig 8 legend here (appearing on lines 248-254):

   > Fig 8: Relationship between pre-test (prior) probability and post-test (posterior) probabilities. a) the machine-learned predictor, b) BNP when greater than 150pg/ml, or c) BNP when greater than 50pg/ml. A positive test is shown in red, and a negative test in green. The diagonal dashed black line represents no change from the pre-test to post-test probability. The vertical dashed black lines represent intermediate to high pre-test probabilities (vertical dashed lines at 30%, 50% and 70%) from left to right. The post-test probabilities were calculated based on a varying pre-test probability, and constant sensitivity, specificity, and corresponding likelihood ratios.

3. Figure 9: This figure is not very clear. Personally, I don't think it is necessary to have a figure to describe the random forest, as it is a very well-known method. I think it is best to describe what setup was used in the Random Forest, such as bootstrapping=True, how many estimators, any limits for the number of trees, leaves etc. Same for the figure of XGBoost in the appendix.

   Thank you, we've removed Figure 9. S10 previously contained the hyperparameter settings for Elastic Net, XGBoost and Random Forest, and we've expanded that to include information on bootstrapping configuration, and to clarify that default hyperparameters settings were used in all other cases. Please see that reproduced below.

Unless otherwise noted in the table, the hyperparameters for the algorithms were set to the default value.

Number	Algorithm	Training Data	Number of Features	Hyperparameters
1	Random Forest	LVEDP≤12 and LVEDP≥20	180	Maximum Tree Depth = 3<br>Minimum Samples Per Leaf = 1<br>Number of Trees = 500<br>Bootstrapping = True
2	Elastic Net	LVEDP≤12 and LVEDP≥20	194	Alpha = 0.003<br>Fit Intercept = True<br>L1_ratio = 0<br>Normalize = False
3	Random Forest	LVEDP≤12 and LVEDP≥20	95	Maximum Tree Depth = 3<br>Minimum Samples Per Leaf = 1<br>Number of Trees = 100<br>Bootstrapping = True
4	Extreme Gradient Boosting	LVEDP≤12 and LVEDP≥25	162	Learning Rate = 0.5<br>Maximum Tree Depth = 3<br>Minimum Child Weight = 3<br>Number of Trees = 100<br>Regularization Alpha = 0.1
5	Extreme Gradient Boosting	LVEDP≤12 and LVEDP≥25	194	Learning Rate = 0.3<br>Maximum Tree Depth = 7<br>Minimum Child Weight = 3<br>Number of Trees = 100<br>Regularization Alpha = 0.5
6	Extreme Gradient Boosting	LVEDP≤12 and LVEDP≥25	134	Learning Rate = 0.5<br>Maximum Tree Depth = 7<br>Minimum Child Weight = 5<br>Number of Trees = 100<br>Regularization Alpha = 0.1
7	Elastic Net	LVEDP≤12 and LVEDP≥25	194	Alpha = 0.003<br>Fit Intercept = True<br>L1_ratio = 0<br>Normalize = True
8	Extreme Gradient Boosting	LVEDP≤12 and LVEDP≥25	152	Learning Rate = 0.3<br>Maximum Tree Depth = 5<br>Minimum Child Weight = 3<br>Number of Trees = 100<br>Regularization Alpha = 0.3
9	Elastic Net	LVEDP≤12 and LVEDP≥25	122	Alpha = 0.01<br>Fit Intercept = True<br>L1_ratio = 0.1<br>Normalize = True
10	Random Forest	LVEDP≤12 and LVEDP≥25	179	Maximum Tree Depth = 3<br>Minimum Samples Per Leaf = 1<br>Number of Trees = 500<br>Bootstrapping = True

11	Random Forest	LVEDP≤12 and LVEDP≥25	89	Maximum Tree Depth = 7<br>Minimum Samples Per Leaf = 1<br>Number of Trees = 50<br>Bootstrapping = True
12	Extreme Gradient Boosting	LVEDP≤12 and LVEDP≥25	119	Learning Rate = 0.3<br>Maximum Tree Depth = 7<br>Minimum Child Weight = 3<br>Number of Trees = 10<br>Regularization Alpha = 0.3
13	Elastic Net	LVEDP≤12, LVEDP≥25, healthy subjects	122	Alpha = 0<br>Fit Intercept = False<br>L1_ratio = 0.1<br>Normalize = False
