# Supplementary material for: Multicenter validation of a machine learning phase space electro-mechanical pulse wave analysis to predict elevated left ventricular end diastolic pressure at the point-of-care
Source: PLoS One. 2022 Nov 15;17(11):e0277300. doi: 10.1371/journal.pone.0277300 (PMC9665374; doi:10.1371/journal.pone.0277300)
Supplement: Attachment — Submitted filename: Author Response To Reviewer_SEPT 24.docx [file pone.0277300.s013.docx]

Dear Reviewer #2,

Thank you for your thorough review and valuable comments. We appreciate and agree with your concern on overfitting and the corresponding risks. We view the recognition of this problem as one of the most significant in the field of machine learning. While we cannot definitively prove that overfitting has not occurred, we believe that the application of our various methods acts to sufficiently mitigate the of likelihood of its occurrence.

Specifically, our most critical control for overfitting was the single evaluation on a test dataset separated from the development set by enrollment date. Further, the probability of our model achieving the performance of 0.81 by chance on a dataset of that size (which could be the case if our development set was overfit) is low, as shown by the comparison between the curve and the random predictor that lies along the diagonal of the ROC space (p<2E-16 by DeLong’s test).

We also implemented a series of additional controls to increase the chance that our algorithm would generalize to an unseen dataset:

- Use of modelling methodologies with regularization and penalty terms that are well-suited to high-dimensional spaces.
- Cross-validation model development with careful monitoring of fold-to-fold standard deviations
- Careful choice of models to ensemble based on hyperparameter stability

Below we provided detailed explanations of our methodologies to address your specific concerns, and we hope that we have addressed them to your satisfaction. Our responses appear below in **bold**, provided in *italics* where applicable in the response, and modified manuscript text in track changes*.*

Sincerely,

Sanjeev Bhavnani MD

Specific Comment #1: In order to clarify our imposed safeguards in reference to overfitting, we have revised the text on lines 350-355.

*The use of an ensemble, as is the case, does not increase the likelihood of overfitting but rather mitigates it by reducing the dependence on a single model. The methods employed to avoid overfitting include the use of cross-validation within the development data, using simple models with regularization and penalty terms, and testing the performance of the model on unseen data (only doing so only once). With respect to the models in particular; first, each model is exposed to an average of 149 features (with a range of 89-194, supplement section 10).*

a. Generally, a good ratio between sample size and parameters to avoid overfitting is somewhere above 10 to 1. This means for an average of 149 features, you would need about 1490 subjects. If I am not mistaken, the maximum number included in this study is 684 post exclusions. It is true that the tree-based methods (Random Forest and XGB) and the elastic net can help reduce the risk of overfitting, but it does not mean you will not overfit because you are using these models. I have seen many overfitting cases when people use those models. I do see the authors trying to manage overfitting by limiting the maximum depth, but there are other parameters as well that need to be looked into.

**Author Response: To clarify the number of subjects – the N=684 that you referenced is the validation cohort, while the models were developed on the N=1272 development cohort (and specifically, each model using a subset of the available cohort, as shown in supplement section 10). With respect to your other comments in this section, we hope that they will be addressed in the responses to your other questions.**

b. One of my additional questions here is how did you choose the hyperparameters for your tree-based models? What type of tunning was performed?

**Author Response: The possible hyperparameter values are shown below for XGB and Random Forest and were chosen to provide a range over the conservative values of each hyperparameter, with some exponential changes where appropriate to ensure coverage. Each combination of hyperparameters settings was trained within the development dataset only using cross-validation (recall that the ensemble was locked, and the validation data evaluated only once).**

| **XGB Hyperparameters** | |
| --- | --- |
| Hyperparameter | Possible Values |
| Learning Rate | 0.1, 0.3, 0.5 |
| Maximum Tree Depth | 2, 3, 5, 7 |
| Minimum Child Weight | 1, 3, 5 |
| Number of Trees | 5, 10, 50, 100 |
| Regularization Alpha | 0.1, 0.3, 0.5 |

| **Random Forest Hyperparameters** | |
| --- | --- |
| Hyperparameter | Possible Values |
| Maximum Tree Depth | 2, 3, 5, 7 |
| Minimum Samples Per Leaf | 1, 25, 50, 100 |
| Number of Trees | 5, 10, 50, 100, 500 |

**A heuristic stability assessment was performed during the selection of each model. Specifically, for a given model, the cross-validation performance of each “neighboring” hyperparameter specification was compared with that of the model under consideration, and the difference evaluated, with the goal of determining whether that region of the hyperparameter space generated similar cross-validation performances as a measure of stability. To illustrate the method, consider the toy example below using XGB, which assumes that XBG only requires the learning and minimum child weight hyperparameters. Varying these parameters each generates an AUC value, as described in the following table.**

|  | Minimum Child Weight | | |
| --- | --- | --- | --- |
| Learning Rate | 1 | 3 | 5 |
| 0.1 | AUC _0.1,1_ | AUC _0.1,3_ | AUC _0.1,5_ |
| 0.3 | AUC _0.3,1_ | AUC _0.3,3_ | AUC _0.3,5_ |
| 0.5 | AUC _0.5,1_ | AUC _0.5,3_ | AUC _0.5,5_ |

**The resultant AUC values can be used to assess the stability of learning rate = 0.3 and minimum child weight = 3 in the following manner. Should the stability value be close to zero, then that indicates that the performance at that specific learning rate and minimum child weight is relatively insensitive to changes to adjacent hyperparameter values.**

$$Stability= {AUC}_{0.3,3}- \frac{1}{8}\left( {AUC}_{0.1,1}+{AUC}_{0.3,1}+ {AUC}_{0.5,1}+ {AUC}_{0.1,3}+ {AUC}_{0.5,3} {+ AUC}_{0.1,5}+ {AUC}_{0.3,5}+ {AUC}_{0.5,5} \right)$$

**The extension of the methodology is straightforward to higher dimensions of hyperparameters, where the configuration of interest is compared to each of its neighbors in that high-dimensional space, which are found based on changing a single hyperparameter by one step in the assessed range of that hyperparameter.**

**Finally, please note that this is a heuristic that we used to guide our selection of the hyperparameters settings, and we do not claim that it is an optimal strategy. For instance, a limitation of this heuristic is the uneven spacing between hyperparameter settings – though we viewed this as necessary to explore the hyperparameter space, it does result in nonuniform distance between neighbors. A possible future extension of this method could correct for that nonuniform distance.**

c. Similarly, what type of tunning was conducted for the elastic net model? What makes you choose 0.003 or 0.01 for alpha?

**Author Response: Please see the explored hyperparameter space for Elastic Net in the table below. The same hyperparameter stability assessment performed for Random Forest and XBG (as described above in b.) was also performed for Elastic Net.**

| **Elastic Net Hyperparameters** | |
| --- | --- |
| Hyperparameter | Possible Values |
| Alpha | 0, 0.003, 0.01, 0.1, 0.5 |
| Fit Intercept | True, False |
| L1 Ratio | 0, 0.01, 0.1, 0.5 |
| Normalize | True, False |

d. Also, an AUC of 0.81 from the validation does not prove that overfitting did not occur since it is just one data point. One can easily argue if there is no overfitting, you could have an AUC of 0.95.

**Author Response: The point is well taken. Please see discuss on page one regarding AUC.**

e. In summary, it is still not very clear how the hyperparameters were chosen for each model. And it is unfair for the authors to conclude that the overfitting did not occur based on an AUC of 0.81 from validation. Also, please remove the word “high-performing”, as a word like “high” or “low” is subjective and not scientific.

**Author Response: We agree that the terms inferring “high/low” are subjective and the text has been altered accordingly on lines 368-369, as shown below. With respect to the hyperparameter selection, we hope that it is now clear from the response to question 1b.**

*Finally, the ultimate test of overfitting is the performance on unseen blinded data, which yielded an AUC of 0.81.*

Specific Comment #2: Regarding the type of catheter, the authors have replied to Comment #10 that the choice of the catheter was left to the discretion of each cardiologist, which means the device for measuring the ground truth can be different between time and the medical centre. Have the authors analysed the potential impact of this?

**Author Response: The reviewer makes an excellent point that variations in catheter technique could affect the consistency of measurements. Such sources of variability are an important concern. However, since 6-French to 8-French catheters are the primary options for left ventricular catheterization, recorded pressure should vary minimally as a function of catheter selection. Moreover, since a variety of catheter types and sizes are used in clinical practice, specifying a particular catheter type may impair the generalizability of the resultant algorithm.**

Specific Comment #3: Is there a reason that the authors can only provide a list of the families of features used in the study instead of a list containing the actual features?

**Author Response: Describing all of the features was out of scope for this paper, and therefore we provided feature description at the level of the family in the interest of brevity. However, we did provide detailed information on several of the features (Fig 3 and Supplement Section 3) to allow the reader to gain an appreciation for the prediction mechanism. In addition, many of the features are proprietary in nature.**

Specific Comment #4: What type of cross-validation did the authors use? Can they specify it in the paper?

**Author Response: The type of cross-validation used was stratified 5-fold cross-validation repeated for 100 iterations (to vary the train/test folds). Please see the modified text from lines 522-523 below:**

*Each of the 13 models were individually performant based on stratified 5-fold cross-validation repeated for 100 iterations (to vary the train/test folds) within the development data (S11) but represent unique analyses of LVEDP assessment.*
